# Primary pulmonary miliary tuberculosis presenting as immune thrombocytopenia- a rare case report

**DOI:** 10.1186/1471-2334-14-S3-P56

**Published:** 2014-05-27

**Authors:** Ashalata Gannepalli, Sanjay Reddy Podduturi, Bhargavi Krishna Ayinampudi, Ramakrishna Angali

**Affiliations:** 1 Department of Oral and Maxillofacial Pathology, Panineeya Institute of Dental Sciences and Research Centre, Hyderabad, India; 2 Department of Oral Medicine & Radiology, Awadh Dental College and Hospital, Jamshedpur, India

## Background

Tuberculosis (TB) is a multifaceted disease, which may present with a variety of symptoms, sometimes mimicking autoimmune diseases. Immune thrombocytopenic purpura (ITP) is an exceedingly rare manifestation of tuberculous infection.

## Case report

A 42 year old female patient presented with petechiae on palate, tongue and extremities, melena and also developed conjunctival hemorrhage, hemoptysis, occult blood in stool. She had a history of evening rise of temperature since one month, non-productive cough since one week. Physical examination revealed no organomegaly, no lymphadenopathy and detailed investigations were done. Chest radiograph showed fine nodular opacities throughout lung field but tuberculin test and early morning sputum AFB examination for three consecutive days was negative. Hb 9.6gm%, PCV28, TLC4100/cumm, platelets-32000/cumm, ESR 52mm in 1st hour and 94mm in 2nd hour. Ultrasound abdomen was normal, HIV ELISA, dengue antibodies was negative, ANA and RA was positive. Diagnosis of pulmonary miliary tuberculosis was considered and patient was given platelet transfusion, IV corticosteroids, and was on anti tubercular therapy for 6 months. The platelet count was normal within ten days, lung lesions resolved and no antibodies were detected after six months. There was no recurrence of TB or ITP and no complain of joint pains after a follow up of three years.

## Conclusion

ITP is a diagnosis of exclusion and TB should be considered as one of the possibility in its differential interpretation. Little is known on the clinical significance of auto antibodies in TB but the present case highlights the importance of anti tubercular therapy in such cases.

